# The Critical Importance of Autopsy in Diagnosing Acute Idiopathic Pulmonary Hemorrhage of Infancy

**DOI:** 10.1155/crpe/9118262

**Published:** 2026-06-22

**Authors:** Michael Narvey, Deima Alammary

**Affiliations:** ^1^ Department of Pediatrics, Rady Faculty of Health Sciences, University of Manitoba, WN2066 820 Sherbrook St, Winnipeg, R3A1R9, Manitoba, Canada, umanitoba.ca; ^2^ Department of Pediatrics, Queen’s University, 76 Stuart St., Kingston, K7L2V7, Ontario, Canada, queensu.ca

## Abstract

We present the case of a newborn with acute idiopathic pulmonary hemorrhage of infancy. Clinicians should be aware of this possibility in a newborn with acute severe pulmonary hemorrhage. As this is a diagnosis of exclusion, consent for autopsy should be encouraged when this diagnosis is suspected to help better understand the scope of this condition.

## 1. Introduction

Pulmonary hemorrhage (PH) can be life‐threatening in the neonate. The importance of autopsy is highlighted as being critical to establishing the diagnosis and has proven to be both helpful in the later debriefing with staff and for the parents who had closure after the loss of their child.

## 2. Clinical Presentation

A 32‐year‐old primigravida mother presented for induction secondary to being postdates at 41 weeks and 6 days of gestation. She had neither diabetes nor hypertensive disorders and did not consume recreational drugs, alcohol, or tobacco products. Her antenatal testing was negative for HIV, syphilis, chlamydia, varicella, herpes, and Group B streptococcus, and she was immune to rubella. This was an unremarkable pregnancy with no fetal anomalies or concerns with fetal growth or amniotic fluid volume on her antenatal fetal ultrasound assessment. The maternal and family history demonstrated no suspicion of hematological or coagulation disorders for either parent.

She developed a nonreassuring fetal heart rate during induction and a maternal temperature above 38 degrees Celsius with suspicion of chorioamnionitis. Due to heart rate abnormalities, an emergent cesarean section was performed, with meconium noted in the amniotic fluid.

A male infant was born with a birth weight of 4.020 kg and with Apgar scores of 1, 3, 4, 5, and 7 at 1, 5, 10, 20, and 30 min, respectively. His arterial cord blood gas demonstrated a pH of 6.87, pCO2 of 117 mm Hg, HCO_3_ of 12 mmol/L, and base excess of −15. At initial assessment, he had poor tone and no respirations with a heart rate under 100 BPM and received immediate positive pressure ventilation with a rise in heart rate over 100 BPM within 2 min of age. He was intubated for ongoing respiratory depression at 5 min of age and noted to have fresh blood coming from the mouth, nares, and larynx during laryngoscopy. After intubation, he began to make respiratory efforts and neurologically became more active with only mildly increased tone. He received a dose of intramuscular vitamin K after intubation.

He was placed on a conventional mechanical ventilator on 100% FiO_2_ using a PEEP of +8 cm H_2_O and achieved oxygen saturations in the 70%–75% range. Pre‐ and postductal oxygen saturation monitoring revealed a difference of up to 20%, consistent with a diagnosis of persistent pulmonary hypertension. Hypoxic–ischemic encephalopathy was considered, given his depressed state at delivery and cord blood gas, but he did not qualify for therapeutic hypothermia, given his neurological examination and concern that severe pulmonary hypertension and bleeding might both be exacerbated with cooling. Consistent with his neurological status improving, at no time did he have a seizure.

He was given a dose of intratracheal surfactant for treatment of his PH without any demonstrable improvement in oxygen saturation. There was no bleeding from other sites, nor petechiae or purpura, but rather, through his care, bleeding was isolated to PH. At one hour and 38 min of age, a CBC revealed a white blood cell count of 16.9 × 10^9^/L., hemoglobin of 156 g/L, and a platelet count of 171 × 10^9^/L.

At 3 h of age, he had another significant PH through his ETT and was treated with fresh frozen plasma for an INR of 2.7. Due to declining oxygen saturations again to 70%–75%, he was changed to high‐frequency jet ventilation (HFJV), and a second dose of surfactant was ordered for ongoing PH.

After only 4 mL of intratracheal surfactant, another significant PH occurred, preventing completion of the dosing. He was disconnected from the HFJV as the tubing had filled with blood and was given manual PPV. He developed heart rates below 60 BPM, necessitating the start of chest compressions, which lasted 6 min, along with two doses of bolus epinephrine.

Following the resuscitation, his hemoglobin was found to have decreased from 156 to 66 g/L, leading to a blood transfusion of 20 mL/kg packed red blood. With deteriorating oxygen saturations to approximately 40%, attempts were made to oxygenate through increased PEEP to as high as 15 cm H_2_O and to attempt tamponade of the bleeding as well. At the age of 6 h, his pH was 6.51, pCO_2_ was 100 mm Hg, pO_2_ was 42 mm Hg, and HCO3 was 4 mmol/L with a base deficit of 29 on 100% oxygen. He developed pulseless electrical activity shortly after his blood gas was drawn. He received an additional 5 min of chest compressions and one more bolus dose of epinephrine before his family decided to stop resuscitation. At the conclusion of resuscitative efforts, a total of 250 mL of fresh blood has been suctioned from the endotracheal tube.

## 3. Autopsy Report

**FIGURE 1:**
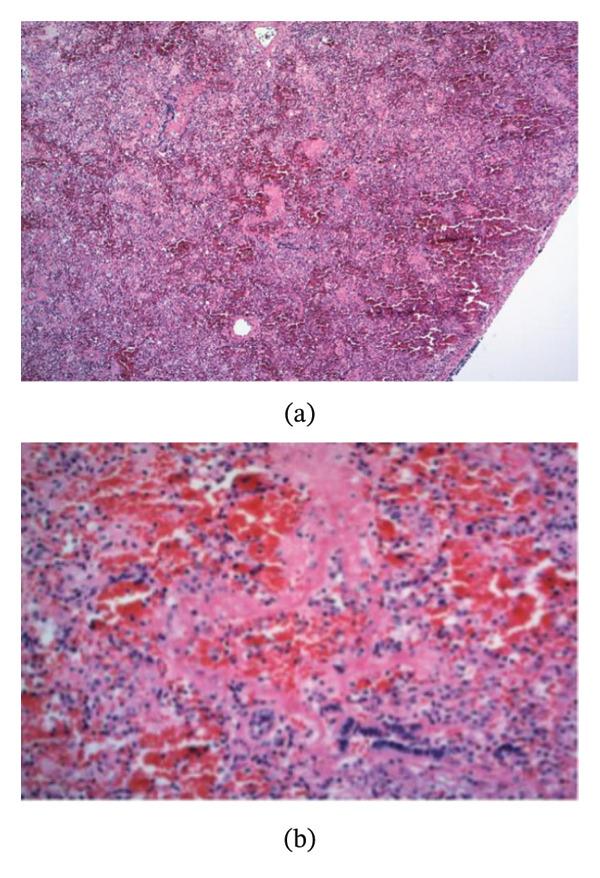
Hematoxylin and eosin‐stained sections at (a) 40X magnification and (b) 200X magnification show massive pulmonary congestion with hyaline membrane formation.

Autopsy revealed massive PH with a resultant combined lung weight of 78.2 g (expected for 41 weeks of gestation: 52.3 ± 16.1 g) [1] as well as bilateral hemothorax (29 and 28 mL in the right and left pleural cavities), respectively. On microscopy, massive intraalveolar congestion and widespread hyaline membrane formation were encountered (Figures 1a and b). Only focal intraalveolar hemosiderin deposition was identified by a Perls Prussian blue iron stain. The interstitium showed only mild–moderate edematous thickening without abnormal cellular infiltration or fibrosis. Viral cytopathic effect was not encountered. The pulmonary vasculature showed moderate arteriolar thickening in keeping with the clinical diagnosis of pulmonary hypertension. There was no evidence of vascular developmental anomaly or capillaritis. No thrombosis was encountered. A postmortem cardiac blood culture yielded no organism growth.

Other significant findings on autopsy were a widely patent (5 mm) ductus arteriosus. There was otherwise no evidence of structural congenital anomaly.

Based on the clinical presentation and lack of explanatory findings for massive PH at autopsy, the infant was diagnosed with acute idiopathic PH of infancy (AIPHI).

## 4. Discussion

The incidence of PH in neonates ranges from 1 to 12 per 1000 live births in the initial days of life, with a mortality rate of 50%. Autopsy data show variable incidences of PH, ranging from 0.86% to 37.3%. The lack of updated epidemiological data, the rarity of the condition, and the absence of diagnostic tools or postmortem histological evaluations contribute to this variability [2]. AIPHI is a rare condition that typically affects otherwise healthy full‐term infants in their first year of life, characterized by the sudden onset of severe respiratory failure with blood in the airways. This diagnosis is elusive in the medical literature, having been reported less than 160 times [3].

PH is distinguished from hyaline membrane disease by the presence of visible blood in the airways, xanthochromic tracheal aspirate or hemoptysis [4]. The clinical criteria for diagnosing AIPHI include a sudden onset of PH in a previously healthy infant aged < 1 year and a gestational age of more than 32 weeks, presenting with a sudden onset of bleeding or visible blood in the airway in addition to severe acute respiratory distress or failure, requiring hospitalization with intubation and mechanical ventilation. CXR or CT scans reveal diffuse pulmonary infiltrates, either unilateral or bilateral [4].

Radiographic findings in AIPHI resemble those of respiratory distress syndrome (RDS) and are distinguished by varying degrees of diffuse alveolar infiltration [4].

As the initial hemoglobin was normal, we suspect that with aeration of the lung and resultant decrease in pulmonary vascular resistance, bleeding ensued from increasing pulmonary blood flow. Hemorrhagic shock ensued purely due to pulmonary bleeding with no other sites of bleeding identified on autopsy. Typical measures to mitigate active bleeding, including intratracheal surfactant and high mean airway pressure to tamponade, failed to control the bleeding. Nitric oxide, while indicated in the presence of this degree of hypoxemia, may have led to further left‐to‐right shunting across the ductus, which may have led to further hemorrhage. As the bleeding occurred within 5 min of birth, shunting through the ductus arteriosus as a primary trigger is highly unlikely, as even extremely preterm infants do not have significant left‐to‐right flow this early. In a lung with a propensity to bleed, however, even a small increase in left‐to‐right flow would have led to the onset of hemorrhaging. The severity of bleeding worsened within the first several hours of life with volume expansion and may have been secondary to distension of blood vessels in the lung, leading to further leakage.

The normal platelet count excludes thrombocytopenia as a cause. Vitamin K‐deficient bleeding would not occur this early, and this patient did receive Vitamin K. While the cord blood gas and neonatal depression might seem to implicate a severe hypoxic–ischemic insult with resultant coagulopathy as an etiology, this is unlikely to be attributable. The unremarkable neurological examination after resuscitation and absence of bleeding from any sites other than the pulmonary source make a coagulopathy from in‐utero hypoxia and ischemia improbable as a cause. He did have a mild perturbation of his coagulation as reflected in an INR of 2.7; however, this was most likely secondary to the significant hemorrhage over the first 3 hours of life and loss of coagulation factors rather than being a primary disturbance of coagulation. Moreover, a primary coagulopathy would not likely have been isolated to PH. Genetic testing was not performed postmortem, but in the absence of a family history of bleeding, an abnormality was not anticipated. This case meets the definition of AIPHI based on the CDC criteria, exclusion of other possibilities above and the autopsy findings excluding hemorrhagic pulmonary conditions such as hemangiomas or arteriovenous malformations as a cause.

The Centers for Disease Control and Prevention established the AIPHI diagnostic criteria in response to clusters observed in Cleveland, Ohio, in the 1990s [5]. 11 cases were identified in Cleveland, Ohio. At presentation, affected neonates had radiographic evidence of diffuse pneumonia and hemoptysis. Since then, some similar cases have been reported from disparate locations in the United States and from other countries [6–8].

There are growing concerns that a missed diagnosis of idiopathic PH may be the cause of unexpected infant death or SIDS [9]. A study suggested criteria for the pathological classification of AIPHI based on a review of other studies, which concludes that AIPHI involves an increase in lung weight; interalveolar bleeding, whether it is a diffuse or localized pattern; the presence of hemosiderin; siderophages, which are typically considered markers of previous bleeding; and hyaline membranes in the absence of an underlying or triggering condition [10]. The authors also felt that some cases of SIDS could be attributable to unrecognized PH.

In this case report, there was an increase in lung weight along with evidence of hyaline membranes and interalveolar hemosiderin, which may indicate that at least one previous bleeding episode occurred in utero.

## 5. Conclusion

Given the rarity of this diagnosis, it is imperative that additional cases be reported in order to better understand the spectrum of this disease. This combination of careful exclusion of other causes of PH, along with findings at autopsy and using the CDC definition for probable causes, leads to the conclusion that this infant died from AIPHI. This case is unusual in the onset of significant bleeding in the minutes after birth and the total volume of blood aspirated from the lung. At 250 mL, this represents a near total loss of the infant’s blood volume over the course of several hours of life, demonstrating the rapidity and severity of blood loss in this condition.

To help with further understanding of this condition, a specific registry for AIPHI may be warranted to eventually shed light on the underlying etiology and risk factors.

## Author Contributions

Dr. Michael Narvey was responsible for the writing of the case report and final review and editing of the manuscript. Dr. Deima Alammary was responsible for the development of the discussion and ongoing review of the manuscript.

## Funding

No funding was received for this study.

## Consent

Personal data processing and informed consent were obtained from the parents of the participant included in the study.

## Conflicts of Interest

The authors declare no conflicts of interest.

## Data Availability

Data sharing is not applicable to this article as no datasets were generated or analyzed during the current study.
